# NGS-Guided Precision Oncology in Breast Cancer and Gynecological Tumors—A Retrospective Molecular Tumor Board Analysis

**DOI:** 10.3390/cancers16081561

**Published:** 2024-04-19

**Authors:** Niklas Gremke, Fiona R. Rodepeter, Julia Teply-Szymanski, Sebastian Griewing, Jelena Boekhoff, Alina Stroh, Thomas S. Tarawneh, Jorge Riera-Knorrenschild, Christina Balser, Akira Hattesohl, Martin Middeke, Petra Ross, Anne-Sophie Litmeyer, Marcel Romey, Thorsten Stiewe, Thomas Wündisch, Andreas Neubauer, Carsten Denkert, Uwe Wagner, Elisabeth K. M. Mack

**Affiliations:** 1 Department of Gynecology, Gynecological Endocrinology and Oncology, University Hospital Gießen and Marburg Campus Marburg, Philipps-University, 35043 Marburg, Germany; sebastian.griewing@uk-gm.de (S.G.); jboekhof@med.uni-marburg.de (J.B.); strohali@staff.uni-marburg.de (A.S.); uwe.wagner@uk-gm.de (U.W.); 2 Institute of Molecular Oncology, Philipps-University, 35043 Marburg, Germany; stiewe@uni-marburg.de; 3 Institute of Pathology, University Hospital Gießen and Marburg Campus Marburg, Philipps-University, 35043 Marburg, Germany; fiona.rodepeter@uk-gm.de (F.R.R.); teply@uni-marburg.de (J.T.-S.); akira.hattesohl@uni-marburg.de (A.H.); anne-sophie.litmeyer2@uk-gm.de (A.-S.L.); marcel.romey@uni-marburg.de (M.R.); carsten.denkert1@uni-marburg.de (C.D.); 4 Department of Hematology, Oncology and Immunology, University Hospital Gießen and Marburg Campus Marburg, Philipps-University, 35043 Marburg, Germany; thomas.tarawneh@staff.uni-marburg.de (T.S.T.); jorge.riera-knorrenschild@med.uni-marburg.de (J.R.-K.); petra.ross@staff.uni-marburg.de (P.R.); neubauer@staff.uni-marburg.de (A.N.); elisabeth.mack@staff.uni-marburg.de (E.K.M.M.); 5 Practice for Internal Medicine, Hematology and Internal Oncology, 35043 Marburg, Germany; drbalser@gmx.de; 6 Comprehensive Cancer Center Marburg, University Hospital Gießen and Marburg Campus Marburg, Philipps-University, 35043 Marburg, Germany; middeke@med.uni-marburg.de (M.M.); thomas.wuendisch@uk-gm.de (T.W.)

**Keywords:** precision oncology, next-generation sequencing, breast cancer, gynecological tumors, molecular tumor board

## Abstract

**Simple Summary:**

Identifying the mutational landscape of tumors using next-generation sequencing (NGS) has become substantially more common over the past decade, especially in patients with advanced tumors. However, there is still limited real-world evidence for the clinical benefits of NGS-guided precision oncology. This retrospective analysis of breast and gynecological cancer patients referred to our center’s multidisciplinary Molecular Tumor Board revealed that treatment recommendations were provided to 63.3% of patients, of whom 29.1% received molecular-matched treatment resulting in significantly prolonged progression-free survival. Commonly altered genes included *TP53*, *PIK3CA*, *BRCA1/2*, and *ARID1A*. Overall, NGS-guided precision oncology using panel diagnostics demonstrated improved clinical outcomes in a subset of patients with breast and gynecological cancers in a real-world setting.

**Abstract:**

**Background:** Precision oncology treatments are being applied more commonly in breast and gynecological oncology through the implementation of Molecular Tumor Boards (MTBs), but real-world clinical outcome data remain limited. **Methods:** A retrospective analysis was conducted in patients with breast cancer (BC) and gynecological malignancies referred to our center’s MTB from 2018 to 2023. The analysis covered patient characteristics, next-generation sequencing (NGS) results, MTB recommendations, therapy received, and clinical outcomes. **Results:** Sixty-three patients (77.8%) had metastatic disease, and forty-four patients (54.3%) had previously undergone three or more lines of systemic treatment. Personalized treatment recommendations were provided to 50 patients (63.3%), while 29 (36.7%) had no actionable target. Ultimately, 23 patients (29.1%) underwent molecular-matched treatment (MMT). Commonly altered genes in patients with pan-gyn tumors (BC and gynecological malignancies) included *TP53* (*n* = 42/81, 51.9%), *PIK3CA* (*n* = 18/81, 22.2%), *BRCA1/2* (*n* = 10/81, 12.3%), and *ARID1A* (*n* = 9/81, 11.1%). Patients treated with MMT showed significantly prolonged progression-free survival (median PFS 5.5 vs. 3.5 months, *p* = 0.0014). Of all patients who underwent molecular profiling, 13.6% experienced a major clinical benefit (PFSr ≥ 1.3 and PR/SD ≥ 6 months) through precision oncology. **Conclusions:** NGS-guided precision oncology demonstrated improved clinical outcomes in a subgroup of patients with gynecological and breast cancers.

## 1. Introduction

Next-generation sequencing (NGS) enables the rapid, cost-effective characterization of complex mutational landscapes of tumors in a clinical setting [1]. The preferred NGS approach for clinical diagnostics to date involves targeted gene panels, allowing for the detection of a broad spectrum of well-characterized genomic alterations including short structural variants (SSVs), copy number alterations (CNAs), translocations, and fusions in multiple genes [2]. However, precision oncology has also become feasible due to the rapid expansion of treatable gene aberrations and predictive biomarkers in recent years, especially in lung cancer and gynecological oncology [3,4,5,6]. Notably, recent data from the UK 100,000 Genomes Project, published in January 2024, provided whole-genome sequencing (WGS) information for 13,880 solid tumors. The findings revealed that clinically relevant mutations were present in 20–49% of gynecological cancers, including breast invasive carcinoma, ovarian high-grade serous carcinoma, and uterine endometrial carcinoma. By contrast, other cancers, such as pancreatic, prostate, esophageal, and stomach adenocarcinomas, exhibited clinically relevant mutations in less than 20% of cases [7].

The availability of new precision oncology therapies in breast and gynecological oncology has increased rapidly in recent years, leading to more significant survival benefits from biomarker-directed therapy [8]. In particular, poly (ADP-ribose) polymerase (PARP) inhibitors have gained approval for treating *BRCA*-mutated or homologous recombination-deficient (HRD) epithelial ovarian cancer, as well as germline *BRCA*-mutated breast cancer (BC) through mutation targeting [9,10,11,12,13]. In addition, the FDA has approved tumor-agnostic therapy with pembrolizumab for microsatellite instability-high (MSI-H), mismatch repair-deficient (MMRd), or tumor mutational burden-high (TMB-H) metastatic tumors. TMB-H and MSI-H serve as predictive factors for a positive response to checkpoint blockade, especially in solid cancers like uterine, cervical, and vulvar cancers [14,15,16,17]. In 2023 alone, for example, the FDA greenlit elacestrant for *ESR1*-mutated BC, extended the approval of sacituzumab govitecan for HR-positive and Her2-negative BC, and approved the pan-AKT inhibitor capivasertib for HR-positive, Her2-negative advanced, or metastatic BC with one or more *PIK3CA*/*AKT1*/*PTEN* alterations [18].

In contrast to these developments, the SHIVA trial, the first randomized trial analyzing the outcomes of precision oncology, revealed that employing multigene sequencing and molecularly targeted agents beyond their recommended indications did not enhance progression-free survival (PFS) when compared to physician’s choice treatment in heavily pretreated cancer patients [19]. Given that other studies have similarly reported no benefit from precision oncology, the results were perceived as a significant disappointment [20,21,22]. However, it is essential to note that since the recruitment period of the SHIVA trial between 2012 and 2014, there has been a substantial increase in available cancer treatments, coupled with improved access to affordable advanced NGS-based molecular tumor profiling. In line with these advances, recently published papers have demonstrated improved outcomes in patients with breast and gynecological cancers using precision medicine [14,23,24,25].

Importantly, Charo et al. found that breast and gynecological cancer patients who received higher degrees of matched therapy experienced increased overall response rates, progression-free survival, and a tendency towards improved overall survival [14]. However, the results of the SAFIR02-BREAST trial, a prospective randomized study comparing targeted therapies to standard of care (SoC) treatment in patients with metastatic BC, show improved PFS only for genomic alterations classified as level I/II by the European Society of Medical Oncology (ESMO) Scale for Clinical Actionability of Molecular Targets (ESCAT), but not beyond level II [25]. The benefit of precision oncology was therefore limited to cases with high levels of evidence for therapeutic actionability of the alterations identified.

In this retrospective study, we aimed to assess NGS-guided precision oncology in clinical practice for breast and gynecological cancers, examining patient characteristics, tumor-specific genetic alterations, therapy recommendations, and outcomes in order to contribute additional real-world data and assess the clinical benefit of NGS-guided precision oncology.

## 2. Materials and Methods

### 2.1. Study Cohort and Workflow

All patients with BC or gynecological malignancies for whom extended molecular diagnostics was recommended by the Breast and Gynecological Tumor Board from August 2018 to August 2023 and who consented to NGS analysis were included in this retrospective single-center analysis. No general exclusion criteria were applied based on age, known genetic alterations, or patient performance status at the time of referral, although recommendations for referral to the MTB were in accordance with the consensus patient characteristics of the German Network for Personalized Medicine. Patients with advanced cancers eligible for extended molecular testing should have completed several lines of approved standard therapies, have a further life expectancy of at least 6 months, and be willing and able to receive further treatment including clinical trials and/or off-label therapies [26]. Approval for this investigation was granted by the Ethics Committee of the Faculty of Medicine of Philipps University, Marburg (RS 23/306). The extended molecular tests were performed after obtaining patients’ written consent for NGS diagnostics and the findings subsequently presented to a multidisciplinary MTB, comprising clinical oncologists, pathologists, bioinformaticians, human geneticists, organ specialists (e.g., gynecologists), and scientists with expertise in molecular diagnostics. After discussion by the MTB, cases with MTB recommendations were re-evaluated in the specialized gynecological tumor board.

### 2.2. Tissue Samples and DNA Extraction

DNA and RNA were extracted from FFPE material in accordance with established protocol; the process has already been described in detail in previous publications [27]. Briefly, tumor cells were isolated from formalin-fixed paraffin-embedded (FFPE) tissue slices obtained from biopsies or surgical specimens through microdissection. DNA was extracted for the VariantPlex™ assay using the Maxwell RSC 48 system (Promega, Madison, WI, USA) with the Maxwell RSC FFPE Plus DNA Kit (AS1720). For the FusionPlex™ assay, RNA was extracted using the RSC RNA FFPE Kit (Promega, AS1440). Quantification of nucleic acids was performed using a Quantus Fluorometer with the QuantiFluor ONE dsDNA System (Promega) or a Qubit device (Thermo Fisher, Waltham, MA, USA), utilizing dsDNA HS or RNA HS assays as appropriate.

### 2.3. Library Preparation and Next Generation Sequencing

Target enrichment for next generation sequencing was based on the anchored multiplex PCR-method, which has been described in detail previously [28] and has been implemented in commercially available DNA- and RNA-based sequencing panels. DNA libraries for the detection of short structural variants (SSVs) (single-nucleotide variants (SNVs), insertions/deletions (INDELs)), and copy number alterations (CNAs) were generated using the VariantPlex™ Solid Tumor (ST) panel (67 genes, Supplementary Table S1) or (from Q2/2023) the VariantPlex™ Pan Solid Tumor (PST) panel (185 genes, Supplementary Table S2) (ArcherDX/IDT, Boulder, CO, USA) in accordance with the manufacturer’s guidelines. For fusion transcript (FT) detection, RNA libraries were generated using the FusionPlex™ Solid Tumor panel (53 genes, Supplementary Table S3) or (from Q4/2022) the FusionPlex™ Pan Solid Tumor panel (137 genes, Supplementary Table S4) (ArcherDX/IDT, Boulder, CO, USA) as recommended by the manufacturer. Sequencing was performed on Illumina MiSeq, MiniSeq, or NextSeq 550 Dx Instruments (Illumina, San Diego, CA, USA) with read lengths of 2 × 151 bp.

### 2.4. Analysis of Genetic Variants

Raw reads obtained from the VP and FP assays were analyzed using the Archer Analysis pipeline versions 6.1 to 7.3 (ArcherDX/IDT, Boulder, CO, USA), either on a cloud-based client (Archer Unlimited) or a local server, utilizing default settings. Fusions were only considered if 10 or more primer start sites supported the event. Variant calling was carried out on a virtual panel of 77 genes that were selected based on their clinical actionability as designated by the Oncology Knowledge Base OncoKB. During variant calling, modifications identified by the software were refined based on criteria including a coverage of more than 100× and a variant allele frequency (VAF) of at least 5% as described previously [27] (Supplementary Table S7). CNAs were called if more than 3 gene-specific primers per gene defined an alteration.

### 2.5. Variant Interpretation and Therapy Recommendations

All variants identified, including SSVs, CNAs, and FTs that successfully passed the standard filters of the analysis tools, were systematically examined for potential artifacts by qualified scientists, and only those deemed non-artificial were assessed for clinical significance. Variant classification was based on the current literature and information from publicly available databases, such as OncoKB [29], CIViC [30], JAX CKB [31], and ClinVar [32,33], whereby the most current version was used in each case. Variants were classified as per the recommendations of the Variant Interpretation for Cancer Consortium (VICC-SOP), and likely pathogenic and pathogenic variants (Class 3–5) were assessed for actionability by the clinical MTB Team by searching the literature, the indicated databases, and local, national, and international clinical trial registries [34]. The strength of evidence supporting therapeutic strategies was assessed using the European Society of Medical Oncology (ESMO) Scale for Clinical Actionability of Molecular Targets (ESCAT) [35] and the German Cancer Consortium (NCT/DKTK) [36,37] scales. Recommendations for treatment were based on the degree of supporting evidence and the anticipated practicability of clinical implementation. Treatment options within clinical trials were ranked highest if the MTB did not identify exclusion criteria based on publicly available information.

### 2.6. Immunohistochemistry for PD-L1, Her2, and MMRD/MSI and Determination of Tumor Mutation Burden (TMB)

PD-L1 immunohistochemistry was performed on formalin-fixed paraffin-embedded (FFPE) slices using the anti-PD-L1 antibody clone E1L3N (diluted 1:1000, cell signaling) on the BondMax™ staining device (Leica Biosystems GmbH, Wetzlar, Germany). The evaluation of staining intensity and distribution was performed on tumor cells (TPS score), immune cells (IC score), and as a combined score (CPS score) [38].

MMRD/MSI testing involved immunohistochemical staining for mismatch repair proteins MLH1, PMS2, MSH2, and MSH6 (antibodies diluted 1:50, Cell Marque), where the loss of nuclear staining for one or more proteins indicated a mismatch repair deficiency in the tumor.

The Her2 testing followed the published guidelines that were first formulated as part of the approval of trastuzumab by the FDA and then by ASCO/CAP in the Her2 guidelines of 2007 [39], 2013 [40], and 2018 [41] and affirmed in the most recently published guideline, issued in 2023 [42]. The assay was performed with antibody clone 4B5 (“ready to use”) on a Ventana Roche staining device (Ventana were transferred to ISH testing using the VENTANA Her2 DUAL ISH DNA PROBE COCKTAIL Assay (Ventana Medical Systems Inc., Marana, AZ, USA). Determination of TMB Medical Systems Inc., Marana, AZ, USA). Specimens that yielded HER2 IHC scores of 2+ was not included in the NGS panels and was only performed in all cases of endometrial cancer and selected cases by an external laboratory using TruSight Oncology 500 (TSO500) assay (Illumina, San Diego, CA, USA) as recommend by the MTB.

### 2.7. Outcome and Clinical Data Assessment

Clinical outcomes were monitored to assess tumor response to the prescribed therapies, and the analysis was conducted by calculating progression-free survival (PFS) using local tumor documentation and the electronic patient record. Therapeutic response was assessed based on the revised RECIST 1.1 criteria [43]. PFS was determined from the initial day of treatment with the recommended matched therapy (or unmatched therapy/physician’s choice) until disease progression or death. The PFS ratio (PFSr) was calculated as time to progression from matched or unmatched therapy initiation (PFS2) divided by time to progression associated with the last prior systemic therapy (PFS1) [44]. A PFSr greater than 1.3 was considered indicative of improved PFS with matched therapy [24,45,46]. We considered a PFSr ≥ 1.3 and a SD ≥ 6 months as a major clinical benefit.

### 2.8. Statistical Analyses

PFS in patients treated with MTB-recommended therapies and those treated with physician’s choice therapies was compared using Kaplan–Meier analysis (Log-rank test) using GraphPad Prism software, Version 10.1.1 (270), 21 November 2023 (GraphPad Software Inc., San Diego, CA, USA). Sample size or power calculations were not performed due to the retrospective nature of this study.

## 3. Results

### 3.1. Study Population and MTB Workflow

**Figure 1:**
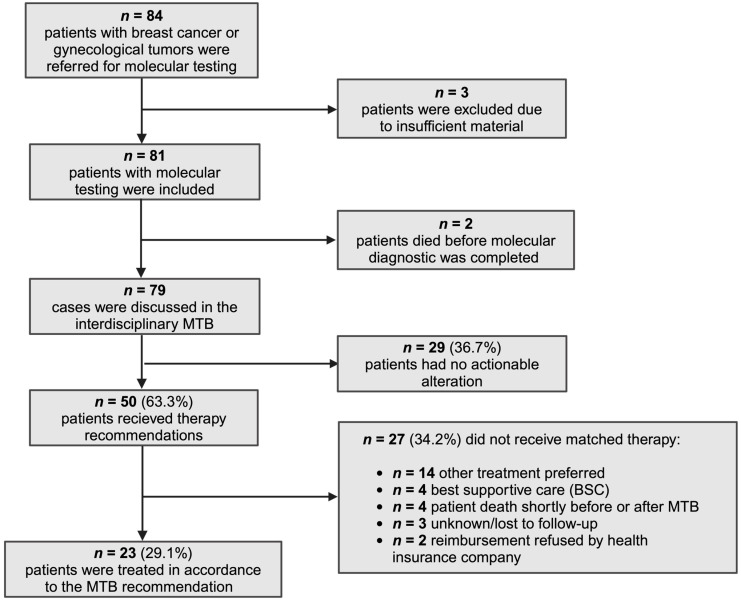
Study population. Extended molecular diagnostics were indicated in 84 cases but could not be performed in three patients due to a lack of tumor material. Seventy-nine patients were discussed in the multidisciplinary MTB and recommendations were made in a total of 50 cases. Twenty-three patients finally received matched therapies.

From August 2018 to August 2023, 84 patients with breast cancer or other gynecological malignancies were referred to our MTB for advanced molecular diagnostics. The advanced molecular diagnostics encompassed a 67-, or 185-gene NGS panel designed for detecting short structural variants (SSVs) and copy number alterations (CNAs), along with a 53- or 137-gene fusion panel. Immunohistochemistry for microsatellite instability, Her2 and PD-L1 expression as well as determination of TMB in selected cases complemented the NGS analysis. Three patients were excluded from our analyses due to insufficient tissue for diagnostics, and two patients died before the conclusion of molecular analyses. In total, 79 patients were discussed in the MTB, with 50 patients (63.3%) receiving personalized therapeutic suggestions, while no actionable target was identified in the remaining 29 (36.7%). Ultimately, 23 patients (29.1%) underwent molecular-matched treatment (MMT). However, 27 patients (34.2%) did not receive MMT, either due to the physician’s preference for other therapy regimens (14/27), the decision for best supportive care (BSC) (4/27), rejection of costs by health insurance companies (2/27), deaths occurring shortly after the MTB (4/27), or loss to follow-up (3/27) (Figure 1).

### 3.2. Characteristics of Patients Undergoing NGS Analysis

Our retrospective analysis included 81 patients with breast cancer or gynecological malignancies for whom advanced molecular diagnostics were recommended. The median age at the time of referral was 59 years. The majority of patients (77.8%) had metastatic disease, even though 59 patients (72.8%) had initially undergone treatment with curative intent and subsequently relapsed or progressed to the metastatic stage. A total of 22 patients (27.2%) already had metastatic disease at the time of diagnosis. The predominant cancer type among patients was breast cancer (44.4%), followed by ovarian cancer (25.9%) and uterine sarcomas (7.4%). More than 50% of all patients had received three or more lines of systemic treatment before MTB discussion (Table 1).

### 3.3. Molecular Testing and Therapeutic Suggestions

**Figure 2:**
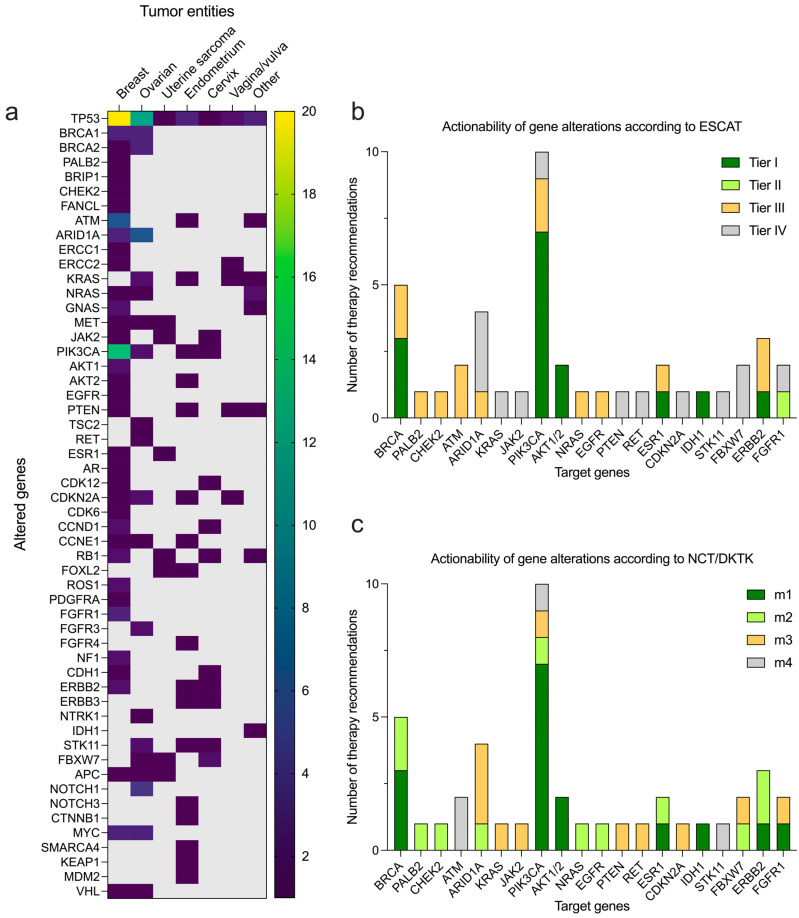
Genetic alterations (SSVs and CNAs) in *n* = 81 tumors identified by targeted sequencing assays. (**a**) List of altered genes stratified by tumor entities. Most common actionable gene alterations classified according to ESCAT (**b**) and NCT/DKTK (**c**) scales for actionability of genetic alterations based on supporting evidence.

Complete NGS diagnostics (FP + VP) were performed in 81 patients. The most commonly altered genes in 36 patients with breast cancer were TP53 (*n* = 20/36, 55.6%), PIK3CA (*n* = 14/36, 38.9%), ATM (*n* = 6/36, 16.7%), and BRCA1/2 (*n* = 4/36, 11.1%). In 45 patients with gynecological cancers the most commonly altered genes were TP53 (*n* = 22/45, 48.9%), ARID1A (*n* = 6/45, 13.3%), BRCA1/2 (*n* = 6/45, 13.3%), and KRAS (*n* = 5/45, 11.1%) (Figure 2a and Supplementary Table S5). Commonly altered genes in all 81 molecular-tested patients with pan-gyn tumors (BC and gynecological malignancies) included TP53 (*n* = 42/81, 51.9%), PIK3CA (*n* = 18/81, 22.2%), BRCA1/2 (*n* = 10/81, 12.3%), and ARID1A (*n* = 9/81, 11.1%). The most frequently observed PIK3CA mutations included the targetable H1047R, E542K, and E545K hotspot mutations as well as PIK3CA gene amplifications. The corresponding levels of evidence for therapeutic actionability according to the ESCAT and NCT/DKTK scales were highest for the PIK3CA and BRCA mutations identified (mainly Tier I or m1, respectively). For the remaining alterations identified, the evidence level was mainly Tier III or m2, respectively (Figure 2b,c).

A total of 14 patients had gene fusions, whereby selected fusions with precisely defined breakpoints are summarized with their relevance in Table 2. Particularly well-characterized gene fusions, such as NTRK-ETV6 in patients with juvenile secretory carcinoma of the breast (JSCV) with a rationale for tumor-agnostic treatment using an NTRK inhibitor (entrectinib), TRIM24-BRAF in VMM with a molecular rationale for MEK inhibitor (trametinib), as well as FGFR2-TACC2 with a therapeutic rationale for an FGFR inhibitor (pemigatinib), were identified in our cohort (Table 2). The very rare KIF5B-RET gene fusion, previously found only in a few breast cancer patients, can be treated with the FDA-approved targeted RET inhibitor selpercatinib. In general, KIF5B-RET fusions are most commonly oncogenic drivers of non-small-cell lung cancers (approx. 1–2% of all cases) and can be treated successfully with selpercatinib [47]. However, this molecularly stratified recommendation of the MTB could not be applied, as the patient died a few days after the MTB. In addition, we have described the SYN2-PPARG gene fusion in VPDC and the YAP1-MAML2 fusion in UUC for the first time in the literature. The detection of BCOR2-ZC3H7B fusion led to the very rare diagnosis of the highly aggressive subtype of high-grade ESS with BCOR2-ZC3H7B fusion (Table 2).

### 3.4. Outcomes with MTB Recommended Therapy

**Figure 3:**
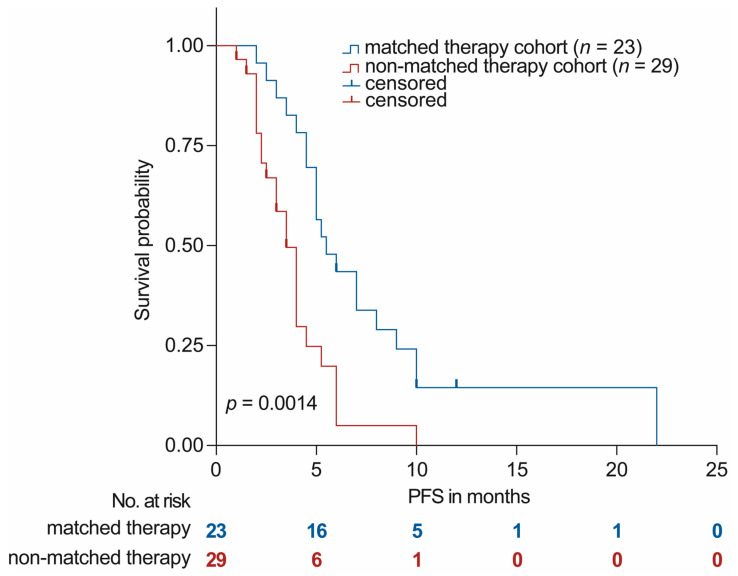
Outcome of patients after molecular profiling and discussion of cases by the MTB. PFS was assessed in patients treated as per the MTB recommendation (*n* = 23) and compared to patients receiving other systemic treatments based on physicians’ choice (*n* = 29). Patients without progression until the end of the observation period and with loss of follow-up were censored. The *p*-value was calculated using a log-rank test.

Of the 81 patients with complete molecular diagnostics, 23 received molecular matched therapy and were followed up for clinical outcome analysis. Among the remaining 58 patients who did not receive molecularly matched therapy, 6 patients died shortly before or after the MTB consultation, 7 patients received BSC, 7 patients remained on the previous line of treatment, and 9 patients were not evaluable due to short-term follow-up (<3 months). In total, 52 out of 81 patients received systemic therapy after MTB discussion and were evaluable for clinical outcomes. The PFS was compared for patients receiving MTB recommended therapy and those treated with other systemic therapies based on the physician’s choice and other recommendations of the Breast and Gynecological Tumor Board using Kaplan–Meier analysis. The PFS in patients treated with matched therapy (recommended by the MTB) (*n* = 23) was significantly prolonged (median PFS 5.5 vs. 3.5 months, *p* = 0.0014, Figure 3). Based on our initial definition of a major clinical benefit, 11 out of 23 evaluable patients achieved a major clinical benefit (PFSr ≥ 1.3 and PR/SD ≥ 6 months) following MTB recommended therapy (Table 3). Thus, among the 81 patients with breast cancer or gynecological malignancies who underwent molecular profiling (11/81, 13.6%) experienced a highly significant clinical improvement (major clinical benefit) through precision oncology. In particular, one patient with a mucinous adenocarcinoma of the appendix (MAAP), which had previously been treated as ovarian cancer, also benefited from the extended molecular diagnostics. By identifying molecular alterations typically found in patients with MAAP (Table 3), the diagnosis was changed accordingly after a new histopathological assessment and the patient was treated with HIPEC and chemotherapy (FOLFOX) as SoC treatment on the MTB’s recommendation and had a PR for 6 months. Furthermore, in a patient diagnosed with primary metastatic breast cancer (mBC), who had previously undergone five lines of systemic therapy, we detected a *PALB2* c.3202-1G>A mutation through extended molecular diagnostics. This mutation was subsequently confirmed as a germline variant, distinct from any germline or somatic *BRCA1/2* sequence variant (Table 3). Following the recommendation of the MTB, the patient received Olaparib treatment, resulting in 22 months of stable disease. This underscores the significance of testing for germline sequence variants in *PALB2*, *CHEK2*, and *RAD51C*, especially considering the clinical potential of PARP inhibitors in mBC, which extends beyond currently approved indications [44,48].

To analyze whether there was heterogeneity between the matched and non-matched therapy cohorts that might influence the outcome of the patients, we performed a cohort analysis based on various factors (tumor stage at presentation, initial therapeutic intent, tumor type, and the number of previous treatment lines). This comparison revealed that similar proportions of patients in both the matched and non-matched therapy cohorts had metastatic (78.3% vs. 75.9%), locally advanced (17.4% vs. 20.7%), and localized tumor stages (4.3% vs. 3.4%). The majority of patients in both the matched and non-matched therapy cohorts had initially undergone treatment with curative intent (69.6% vs. 75.9%), and (30.4 vs. 24.1%) had primary metastatic disease. The predominant cancer type among both cohorts was breast cancer (43.5% vs. 48.3%), followed by ovarian cancer (34.8% vs. 17.2%) and endometrial carcinomas (8.7 vs. 13.8%). In both cohorts, more than 60% of all patients had received three or more lines of systemic treatment before MTB discussion (Table 4 and Supplementary Table S6). In summary, patients in both matched and non-matched therapy cohorts were comparable in terms of their tumor stage, initial therapeutic intention, tumor stage, and number of prior therapies.

## 4. Discussion

NGS-guided precision oncology is increasingly being used to identify treatment options for patients with advanced tumors for whom approved SoC treatments are exhausted. As knowledge on molecular functions of cancer driver genes, the landscape of clinical trials, and availability of drugs for potential off-label use are changing rapidly, MTBs are essential for identifying and ranking therapeutic approaches in individual cases beyond standard treatments [49,50,51]. In our study, 63.3% of all patients discussed by the MTB received recommendations for personalized treatments, which is in line with other recent studies focusing on breast and gynecological tumors. For example, Sultova et al. reported actionable alterations in 41 of 95 patients (43.2%) with BC and gynecological malignancies, whereas Bruzas et al. reported that 63 patients (66.3%) in their mBC cohort were suitable for MMT [23,24]. In cohorts including more extended spectra of different tumor entities, the fraction of patients with actionable alterations varies from 39.5% in Le Tourneau et al. [19] to a maximum of 87% in Walter et al. [52], influenced by the heterogeneity of the study population in terms of their different tumor entities and their diverse mutational landscapes [53,54].

Targeted molecular profiles in our study, encompassing breast cancer and gynecological malignancies (pan-gyn tumors), most frequently revealed mutations in *TP53*, *PIK3CA, BRCA1/2*, and *ARID1A*. This pattern closely resembled molecular data from 2579 pan-gyn tumors in The Cancer Genome Atlas (TCGA) and real-world targeted NGS data from an MTB with 164 pan-gyn tumor patients [14,55]. In particular, both studies showed high rates of alterations in *TP53* (44% of patients in TCGA vs. 54% pan-gyn MTB analysis), *PIKC3CA* (32% vs. 27%), *PTEN* (20% vs. 15%), and *ARID1A* (14% vs. 15%) similar to our observations, while *PTEN* alterations were not among the top four altered genes in our analysis. Finally, only 23 patients (29.1%) in our cohort received molecular-matched treatment (MMT), which is in line with previous studies, although the proportion of patients who received MTT fluctuated greatly for various reasons. For example, Pernas et al., in the SOLTI-1301 AGATA study, and Aftimos et al., in the AURORA study, reported that only 5% and 7% of patients, respectively, received MMT [56,57]. In contrast, the rates of patients who finally received MMT therapy reported by Parker and Walter et al. (42% each) and Bruzas and Fukada et al. (36% and 26%, respectively) were comparable to those in our study [24,52,54,58,59]. In the literature, the reasons given for declining the recommended MTT are the doctor’s or patient’s preference for another therapy, the lack of reimbursement, the unavailability of medication, and progression of the disease, resulting in a poor performance status or death by the time MMT actually became available for a given patient [24,60,61]. In particular, rapid deterioration of Eastern Cooperative Oncology Group (ECOG) status while panel-sequencing is performed has been highlighted as a significant drawback limiting the potential benefits of personalized treatments in other studies, emphasizing the importance of initiating NGS panel sequencing when patients are in an appropriate condition to tolerate MTT [24,62].

Finally, the PFS in pan-gyn tumor patients treated with matched therapy (recommended by the MTB) was significantly prolonged in our study (median PFS 5.5 vs. 3.5 months, *p* = 0.0014). However, of the 81 patients who underwent molecular profiling only a small fraction of 13.6% experienced a major clinical benefit through precision oncology. This small clinical benefit in a highly pretreated population with advanced pan-gyn tumors is congruent with results of the MOSCATO-01 study, a single-center French cancer-screening program on biopsies from metastasis sites in patients with various metastatic malignancies including breast cancer. The primary endpoint of the study was met since 63 out of 948 patients (approximately 7% of all enrolled patients) had a PFSr > 1.3 and therefore benefited from the target drug treatment [63]. In a retrospective analysis of a pan-cancer cohort from our institution, 9.6% of all enrolled patients had a major clinical benefit through precision oncology, and in a study published by Bruzas et al., 13.6% of all included patients with mBC had a clinical benefit from NGS-directed therapy [24,27]. This low percentage seems to remain consistent across various sequencing techniques, even when the number of genes analyzed is increased. Notably, the MTB at our institution currently uses targeted sequencing assays to develop personalized treatment recommendations, although the field of precision oncology is quickly moving towards integrating large gene panels and even system-wide sequencing approaches and proteomics or functional assays into clinical routine [37,64,65,66]. Importantly, findings from the PERMED-01 trial demonstrated that the use of whole-exome sequencing (WES) did not yield more clinically relevant information than gene panels analyzing only a couple of hundred genes [66]. However, studies have indicated that patient clinical outcomes are associated with the level of ESCAT recommendations and adherence to them. Specifically, a study by Andre et al. demonstrated that MTTs improve the PFS with level I/II ESCAT-classified genomic alterations (adjusted HR: 0.41, 90% CI: 0.27–0.61, *p* < 0.001), but not in the entire cohort including recommendations of all ESCAT-levels (adjusted HR: 0.77, 95% CI: 0.56–1.06, *p* = 0.109) [25].

Notwithstanding the many strengths of this study, it is also subject to a number of limitations, which should be noted at this point. Potential biases in interpreting the results of this study stem from its non-randomized design to account for variations in MTT decisions, standardization in molecular analysis, time points to initiate NGS diagnostics, NGS panel types, and clinical follow-up. However, these limitations reflect the real-world nature of this study. Another point is the limited patient cohort, which exhibits significant heterogeneity in terms of tumor entities and prior treatments. Despite the lack of randomization due to the retrospective nature of this study, our cohort analysis revealed that patients in both the matched and non-matched therapy cohorts were comparable in terms of their tumor stage, initial therapeutic intention, and number of prior therapies. Finally, ESMO guidelines advise the use of NGS specifically for patients with ovarian carcinoma, non-squamous NSCLC, prostate carcinoma, and cholangiocarcinoma. Therefore, the application of NGS diagnostics for gynecological patients outside these indications should be evaluated on an individual basis [67].

## 5. Conclusions

This retrospective study may provide further real-world evidence that precision oncology improves the clinical outcome in a small but relevant fraction of highly pretreated patients with advanced gynecological and breast cancers.

## Figures and Tables

**Table 1:** Characteristics of patients undergoing NGS analysis.

	*N*	Percentage (%) or Range
**Total**	81	
	**Sex**	
Female	81	(100)
**Age**	59 (median)	26–84 (range)
	**Tumor stage at presentation**	
Metastatic	63	(77.8)
Locally advanced	16	(19.8)
Localized	2	(2.5)
	**Initial therapeutic intent**	
Primary metastatic, palliative intent	22	(27.2)
Initially curative intent	59	(72.8)
	**Tumor type**	
Breast	36	(44.4)
Ovary	21	(25.9)
Uterine sarcoma	6	(7.4)
Endometrium	4	(4.9)
Cervix	5	(6.2)
Vagina/vulva	4	(4.9)
Others	5	(6.2)
	**Previous lines of treatment**	
0	6	(7.4)
1	13	(16.0)
2	18	(22.2)
3	18	(22.2)
>3	26	(32.1)
	3 (median)	0–14 (range)

**Table 2:** Fusion transcripts and their respective diagnostic or clinical relevance.

Diagnosis	Fusion	Diagnostic or Clinical Relevance
JSCB	*NTRK3*-*ETV6*	Molecular rationale for NTRK inhibitor (entrectinib)
VMM	*TRIM24*-*BRAF*	Molecular rationale for MEK1/2-targeted inhibitor (Trametinib)
UEC	*FGFR2*-*TACC2*	Molecular rationale for FGFR inhibitor (pemigatinib)
IDC	*KIF5B*-*RET*	Molecular rationale for RET-inhibitor (selpercatinib)
VPDC	*SYN2*-*PPARG*	Fusion in VPDC described for the first time in literature, no evidence for direct actionability
UUC	*YAP1*-*MAML2*	Fusion described for the first time in UUC, no evidence for direct actionability
ESS	*BCOR2*-*ZC3H7B*	Led to the very rare diagnosis of high-grade ESS with *BCOR2*-*ZC3H7B* fusion

JSCB: juvenile secretory carcinoma of the breast; VMM: mucosal melanoma of the vulva/vagina; UEC: uterine endometrioid carcinoma; IDC: breast invasive ductal carcinoma; VPDC: poorly differentiated vaginal carcinoma; UUC: uterine undifferentiated carcinoma; ESS: endometrial stromal sarcoma.

**Table 3:** Patients with major clinical benefit (PFSr ≥ 1.3 and PR/SD ≥ 6 months) following MTB recommended therapy.

Diagnosis	Therapeutic Rationale	MTB Recommendation	EL ESCAT	EL NCT/ DKTK	Label	PFS2	PFS1	PFSr	Outcome
VPDC	TMB-H	Pembrolizumab	I-A	m1a	On	7	1	7	SD for 7 months
IDC	*Her2* low (SIS 1+)	Trastuzumab–deruxtecan	I-A	m1a	On	12	4	3	SD for 12 months ^#^
CESC	*Her2* low (SIS 1+)	Trastuzumab–deruxtecan	I-C	m1c	Off	6	3	2	SD for 6 months ^#^
IDC	*PIK3CA* H1047R	Alpelisib + Fulvestrant	I-A	m1a	Off	10	3	3.3	PR for 10 months
IDC	*EGFR* amplification	Cetuximab + Capecitabine	IV-A	m1c	Off	10	2	5	SD for 10 months
SCT *	*RET* Y791F	Cabozantinib	IV-A	m3	Off	10	1	10	SD for 10 months
IDC	g*PALB2* Mutation	Olaparib	III-A	m2a	Off	22	10	2.2	PR for 22 months
HGSOC	*ARID1A* Loss	Pembrolizumab	IV-A	m3	Off	6	1	6	SD for 6 months
OVT	*KRAS* G12D *GNAS* R142H *MET* R540C	Diagnosis changed to MAAP: HIPEC + FOLFOX	n/a	n/a	On	6	3	2	PR for 6 months
UELMS	*ESR1* Y537S	Fulvestant	III-A	m2a	Off	9	2	4,5	SD for 9 months ^#^
IDC	*ESR1* D538G *PIK3CA* H1047R	Aleplisib + Elacestrant	I-A	m1a	Off	8	6	1.3	SD for 8 months

EL: evidence Level; PR: partial remission; SD: stable disease; VPDC: poorly differentiated vaginal carcinoma; TMB-H: tumor mutational burden–high; IDC: breast invasive ductal carcinoma; CESC: cervical squamous cell carcinoma; SCT: steroid cell tumor; HGSOC: high-grade serous ovarian cancer; OVT: ovarian epithelial tumor; MAAP: mucinous adenocarcinoma of the appendix; UELMS: uterine epithelioid leiomyosarcoma; * previously described in [27] Tarwaneh et al.; ^#^ SD until the end of the indicated observation period.

**Table 4:** Patient characteristics within the matched and non-matched therapy cohort.

	Matched Therapy Cohort (*n* = 23)	Non-Matched Therapy Cohort (*n* = 29)
	Total (percentage, %)	Total (percentage, %)
**Tumor stage at presentation**		
Metastatic	18 (78.3)	22 (75.9)
Locally advanced	4 (17.4)	6 (20.7)
Localized	1 (4.3)	1 (3.4)
**Initial therapeutic intent**		
Primary metastatic, palliative intent	7 (30.4)	7 (24.1)
Initially curative intent	16 (69.6)	22 (75.9)
**Tumor type**		
Breast	10 (43.5)	14 (48.3)
Ovary	8 (34.8)	5 (17.2)
Uterine sarcoma	1 (4.3)	3 (10.3)
Endometrium	2 (8.7)	4 (13.8)
Cervix	1 (4.3)	1 (3.4)
Vagina/vulva	1 (4.3)	1 (3.4)
Others	0 (0)	1 (3.4)
**Previous lines of treatment**		
0	2 (8.7)	3 (10.3)
1	4 (17.4)	4 (13.8)
2	2 (8.7)	4 (13.8)
3	5 (21.7)	6 (20.7)
>3	10 (43.5)	12 (41.4)

## Data Availability

Data presented in this research paper will be made available upon request to the corresponding authors.

## Supplementary Materials

The following supporting information can be downloaded at: https://www.mdpi.com/article/10.3390/cancers16081561/s1, Table S1: Target regions of the VariantPlex Solid Tumor Panel (ArcherDx/IDT); Table S2: Target regions of the VariantPlex Pan Solid Tumor Panel (ArcherDx/IDT); Table S3: Target genes of the FusionPlex Solid Tumor Panel (ArcherDx/IDT); Table S4: Target genes of the FusionPlex Pan Solid Tumor Panel (ArcherDx/IDT), Table S5: Genetic alterations in *n* = 81 patients referred to the MTB; Table S6: Patient characteristics within the matched or non-matched therapy cohort; Table S7: (ArcherDx/IDT) filter settings.
